# Association of the uric acid-to-HDL cholesterol ratio with incident type 2 diabetes in community-dwelling older adults in China: a retrospective cohort study

**DOI:** 10.3389/fendo.2025.1720947

**Published:** 2026-01-06

**Authors:** Heping Sun, Shao Zhong, Zhaoxiang Wang, Qianqian Wang, Bing Lu

**Affiliations:** Department of Endocrinology, Affiliated Kunshan Hospital of Jiangsu University, Kunshan, Jiangsu, China

**Keywords:** elderly population, high-density lipoprotein cholesterol, serum uric acid, type 2 diabetes, UHR

## Abstract

**Objective:**

The serum uric acid-to-high-density lipoprotein cholesterol ratio (UHR) is considered an emerging indicator of inflammatory and metabolic disorders. The aim of this research is to assess the link between UHR and the susceptibility to type 2 diabetes among elderly people in Chinese communities.

**Methods:**

A retrospective cohort study was conducted. The participants were elderly non-diabetic individuals who underwent annual health check-ups in the Kunshan community, China, from January 2018 to December 2023. A multivariable Cox proportional hazards model was used to investigate the relationship between baseline UHR levels and the incidence of type 2 diabetes in the elderly population.

**Results:**

After a median follow-up of 3.88 years, 773 type 2 diabetes events were recorded among the 7525 elderly non-diabetic individuals. After adjustment for confounders, each 1–standard deviation (SD) increase in the UHR was associated with a higher risk of type 2 diabetes (HR = 1.156, 95% CI 1.069-1.250, *P* < 0.001), even within the normal UHR range. The relationship between UHR and type 2 diabetes risk is more pronounced in non-obese individuals.

**Conclusion:**

In Chinese community-dwelling elderly individuals, baseline UHR levels are associated with the risk of developing type 2 diabetes. Monitoring UHR levels might help predict and assess the risk of type 2 diabetes.

## 1 Introduction

Diabetes is one of the most serious and widespread chronic illnesses today, leading to dangerous, disabling, and costly complications, and reducing life expectancy (1). The International Diabetes Federation estimated that 536.6 million people worldwide had diabetes in 2021, with about 140.9 million cases in China (2). By 2045, the number of diabetes patients in urban and rural areas is expected to reach 783.2 million (2). The socioeconomic burden of diabetes and its complications is immense and remains significant without any signs of decrease (3).

Elevated serum uric acid (SUA) levels are common in patients with metabolic syndrome and are recognized risk factors for gout, chronic kidney disease, obesity, non-alcoholic fatty liver disease (NAFLD), and cardiovascular diseases (4). Current research indicates that SUA is associated with various pathophysiological mechanisms, including oxidative stress, inflammation, and cell apoptosis (4). On the other hand, high-density lipoprotein cholesterol (HDL-c) is celebrated not only for its anti-atherosclerotic benefits but also for its notable antioxidant and anti-inflammatory properties (5, 6). Moreover, reduced HDL-c levels have also been associated with the pathogenesis of insulin resistance (7–9). The SUA-to-HDL-c ratio (UHR) has recently been introduced as a novel marker for inflammation and metabolism (10). Emerging research underscores that UHR is becoming recognized as a superior predictor for metabolic syndrome, chronic kidney disease, and NAFLD (10–12).

However, to our knowledge, there is currently a lack of research on type 2 diabetes onset associated with UHR in elderly population. To fill this gap, this study uses data from the elderly non-diabetic population in Kunshan community, Jiangsu Province, China, to explore the relationship between UHR and the risk of developing type 2 diabetes.

## 2 Materials and methods

### 2.1 Data source and study population

This retrospective cohort study makes use of population-based electronic health record (EHR) data. Elderly participants were enrolled from January 1, 2018, to December 31, 2023, during complimentary annual health screenings at the Community Health Service Centre in Kunshan City, Jiangsu Province, China. The cohort design details have been previously outlined (13, 14). All participants provided written informed consent for their detailed EHR data to be used. The study protocol received approval from the Institutional Review Board of the First People’s Hospital of Kunshan (2023-03-014-H01-K01). The study included 7525 elderly individuals, excluding those with incomplete EHR data, those diagnosed with type 2 diabetes at baseline, and those younger than 60 years at the time of their initial health examination.

### 2.2 Data collection

The EHR database comprised comprehensive demographic details such as age and gender, along with lifestyle surveys covering smoking habits and alcohol use. Smoking status was divided into former, current, and never smokers, while alcohol consumption was categorized as never, infrequent, regular, or daily. It also included medical history data, noting conditions like hypertension, diabetes, and cardiovascular diseases. Anthropometric data such as height and weight were recorded, alongside routine biochemical test outcomes. Body mass index (BMI) was determined using the formula: BMI = weight (kg)/height (m)^2, with categories defined as normal (<24 kg/m^2), overweight (24–28 kg/m^2), and obese (≥28 kg/m^2). Biochemical tests were conducted on fasting venous blood samples taken after an 8 to 10-hour fast, measuring parameters like fasting plasma glucose (FPG), alanine aminotransferase (ALT), aspartate aminotransferase (AST), blood urea nitrogen (BUN), SUA, serum creatinine (Scr), total cholesterol (TC), triglycerides (TG), HDL-c, and low-density lipoprotein cholesterol (LDL-c). The estimated glomerular filtration rate (eGFR) was calculated using the Chronic Kidney Disease Epidemiology Collaboration (CKD-EPI) formula (15).

### 2.3 Definitions of UHR and type 2 diabetes

UHR, used as the exposure in this study, is derived by comparing SUA (μmol/L) with HDL-c (mg/dL) (7, 16). The primary outcome was incident type 2 diabetes, identified from the integrated community electronic medical record system in Kunshan, which aggregates diagnoses from community health centers and affiliated hospitals. Diabetes was defined as the first occurrence of either an International Classification of Diseases, 10th Revision (ICD-10) diagnosis code for type 2 diabetes (E11-E14), assigned by treating physicians during routine outpatient or inpatient encounters, or a FPG level ≥ 7.0 mmol/L measured during annual health check-ups.

### 2.4 Statistical analysis

In examining baseline characteristics, continuous variables are represented by mean and standard deviation (SD), while categorical variables are depicted as counts and percentages. The Kruskal-Wallis and Chi-square tests were applied to compare UHR quartile groups. A multivariate Cox proportional hazards model was utilized to explore the link between baseline UHR levels (both continuous and categorical) and the development of type 2 diabetes. The Kaplan-Meier method was used to determine the cumulative risk of type 2 diabetes events across UHR quartiles, with the log-rank test employed for group comparisons. A restricted cubic spline (RCS) Cox model was used to assess potential nonlinear relationships between baseline UHR levels and type 2 diabetes risk. The optimal threshold was determined using maximum likelihood estimation. Using time-dependent receiver operating characteristic (ROC) methodology, we assessed the discrimination of the UHR for the risk of diabetes onset over varying follow-up intervals. Subgroup analyses were stratified by age, gender, smoking status, alcohol consumption, BMI, hypertension, cardiovascular diseases, and eGFR, while controlling other confounding factors. All statistical analyses were performed using R software. A two-sided test was conducted, with a *P* value < 0.05 considered statistically significant.

## 3 Results

### 3.1 Baseline characteristics according to UHR quantiles

Involving 7525 subjects with an average age of 66.96 years, of whom 47.95% were male, this study categorized participants into four quartiles (Q1-Q4) based on UHR levels. As shown in **Table 1**, participants in quartiles 2 to 4 were older, more likely to be male, smokers, and alcohol consumers than those in quartile 1 (*P* < 0.001). These quartiles also saw increased hypertension, cardiovascular disease rates, and BMI (*P* < 0.01). Significant rises in ALT, AST, TG, Scr, and SUA were observed (*P* < 0.01), whereas TC, LDL-c, HDL-c, and eGFR decreased (*P* < 0.001). The incidence of type 2 diabetes climbed from 7.02% in quartile 1 to 14.61% in quartile 4, highlighting a significant link between higher UHR levels and type 2 diabetes risk (*P* < 0.001).

**Table 1:** Distribution of baseline characteristics across UHR quartiles.

	Overall (n=7525)	Q1 (n=1880)	Q2 (n=1880)	Q3 (n=1883)	Q4 (n=1882)	*P* value
Age (years)	66.96 ± 4.48	66.58 ± 4.28	66.78 ± 4.23	67.08 ± 4.56	67.39 ± 4.78	<0.001
Gender, n%						<0.001
Female	3917 (52.05%)	1394 (74.15%)	1077 (57.29%)	831 (44.13%)	615 (32.68%)	
Male	3608 (47.95%)	486 (25.85%)	803 (42.71%)	1052 (55.87%)	1267 (67.32%)	
Smoking status, n%						<0.001
Never	5727 (76.11%)	1618 (86.06%)	1479 (78.67%)	1380 (73.29%)	1250 (66.42%)	
Former	277 (3.68%)	37 (1.97%)	53 (2.82%)	81 (4.30%)	106 (5.63%)	
Current	1521 (20.21%)	225 (11.97%)	348 (18.51%)	422 (22.41%)	526 (27.95%)	
Alcohol consumption, n%						<0.001
Never	6000 (79.73%)	1663 (88.46%)	1519 (80.80%)	1424 (75.62%)	1394 (74.07%)	
Infrequently	494 (6.56%)	65 (3.46%)	116 (6.17%)	151 (8.02%)	162 (8.61%)	
Regularly	145 (1.93%)	20 (1.06%)	35 (1.86%)	42 (2.23%)	48 (2.55%)	
Daily	886 (11.77%)	132 (7.02%)	210 (11.17%)	266 (14.13%)	278 (14.77%)	
Hypertension, n%						0.002
No	3819 (50.75%)	995 (52.93%)	995 (52.93%)	930 (49.39%)	899 (47.77%)	
Yes	3706 (49.25%)	885 (47.07%)	885 (47.07%)	953 (50.61%)	983 (52.23%)	
Cardiovascular disease, n%						<0.001
No	7207 (95.77%)	1823 (96.97%)	1811 (96.33%)	1801 (95.65%)	1772 (94.16%)	
Yes	318 (4.23%)	57 (3.03%)	69 (3.67%)	82 (4.35%)	110 (5.84%)	
BMI (kg/m2)	24.50 ± 3.23	23.09 ± 3.13	24.20 ± 3.10	24.97 ± 3.06	25.74 ± 3.03	<0.001
FPG (mmol/L)	5.44 ± 0.65	5.39 ± 0.63	5.38 ± 0.63	5.46 ± 0.66	5.54 ± 0.68	<0.001
ALT (U/L)	19.80 ± 12.74	17.90 ± 13.90	19.02 ± 11.00	20.32 ± 12.34	21.96 ± 13.19	<0.001
AST (U/L)	22.48 ± 10.42	22.34 ± 12.18	22.29 ± 9.69	22.21 ± 9.17	23.10 ± 10.38	0.029
TG (mg/dL)	1.63 ± 1.13	1.18 ± 0.58	1.42 ± 0.68	1.64 ± 0.89	2.29 ± 1.68	<0.001
TC (mg/dL)	184.57 ± 35.58	193.02 ± 34.85	186.79 ± 34.16	181.33 ± 34.12	177.14 ± 37.10	<0.001
HDL-c (mg/dL)	53.60 ± 19.38	68.35 ± 30.06	55.60 ± 9.10	49.50 ± 7.61	40.94 ± 7.78	<0.001
LDL-c (mg/dL)	104.39 ± 30.45	106.24 ± 31.91	107.46 ± 29.10	104.59 ± 28.72	99.27 ± 31.32	<0.001
BUN (mmol/L)	5.70 ± 5.34	5.72 ± 7.19	5.71 ± 7.47	5.64 ± 1.42	5.75 ± 2.22	0.931
Scr (μmol/L)	72.44 ± 20.98	64.82 ± 18.38	70.36 ± 23.93	74.19 ± 15.14	80.39 ± 22.21	<0.001
SUA (μmol/L)	324.03 ± 83.12	244.80 ± 50.98	300.69 ± 49.10	342.47 ± 52.63	408.01 ± 75.15	<0.001
eGFR (ml/min/1.73 m2)	84.49 ± 12.91	88.19 ± 10.99	85.82 ± 11.65	83.61 ± 12.28	80.34 ± 15.05	<0.001
UHR	6.56 ± 2.68	3.71 ± 0.73	5.42 ± 0.40	6.94 ± 0.50	10.18 ± 2.24	<0.001
T2DM, n%						<0.001
No	6752 (89.73%)	1748 (92.98%)	1717 (91.33%)	1680 (89.22%)	1607 (85.39%)	
Yes	773 (10.27%)	132 (7.02%)	163 (8.67%)	203 (10.78%)	275 (14.61%)	

BMI, body mass index; FPG, fasting plasma glucose; ALT, alanine aminotransferase; AST, aspartate aminotransferase; TG, triglyceride; TC, total cholesterol; HDL-c, high-density lipoprotein cholesterol; LDL-c, low-density lipoprotein cholesterol; BUN, blood urea nitrogen; Scr, serum creatinine; eGFR, estimated glomerular filtration rate; SUA, serum uric acid; UHR, serum uric acid-to-high-density lipoprotein cholesterol ratio.

### 3.2 Associations between UHR and incident type 2 diabetes

**Figure 1:**
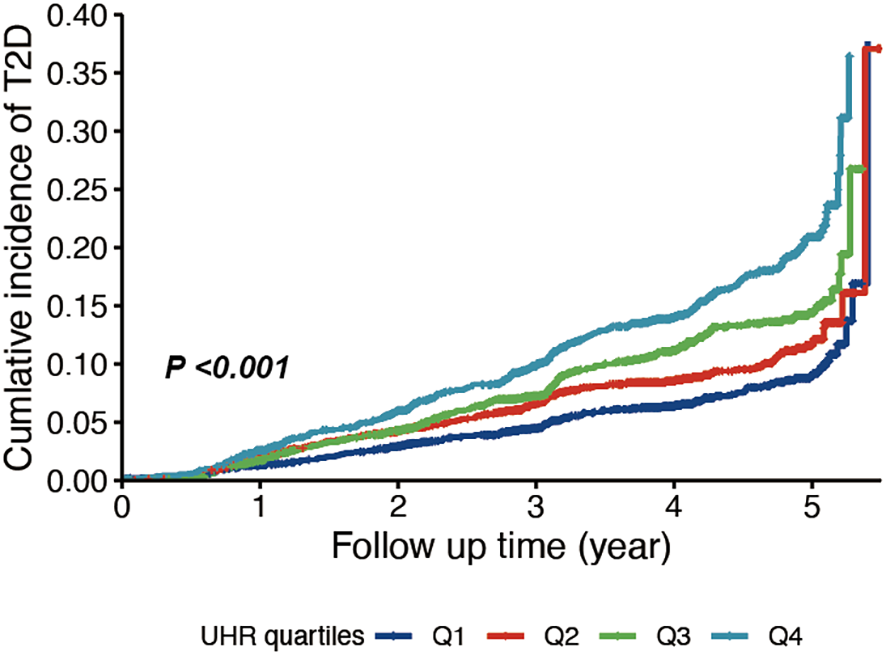
Cumulative incidence of type 2 diabetes by UHR quantiles.

**Figure 2:**
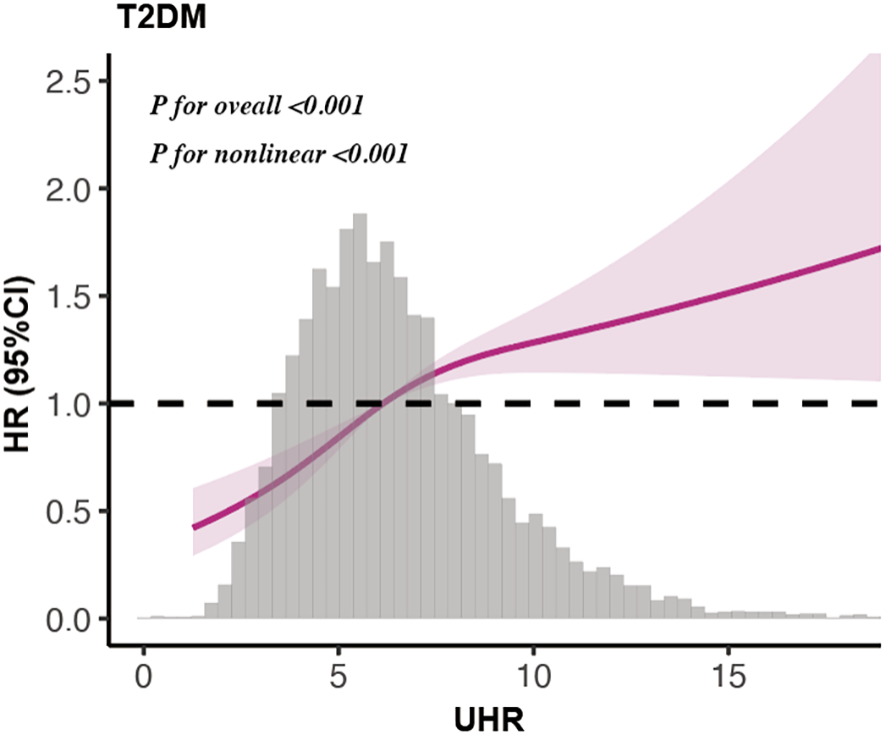
Nonlinear association between UHR and incident type 2 diabetes from Restricted cubic spline Cox models.

**Figure 3:**
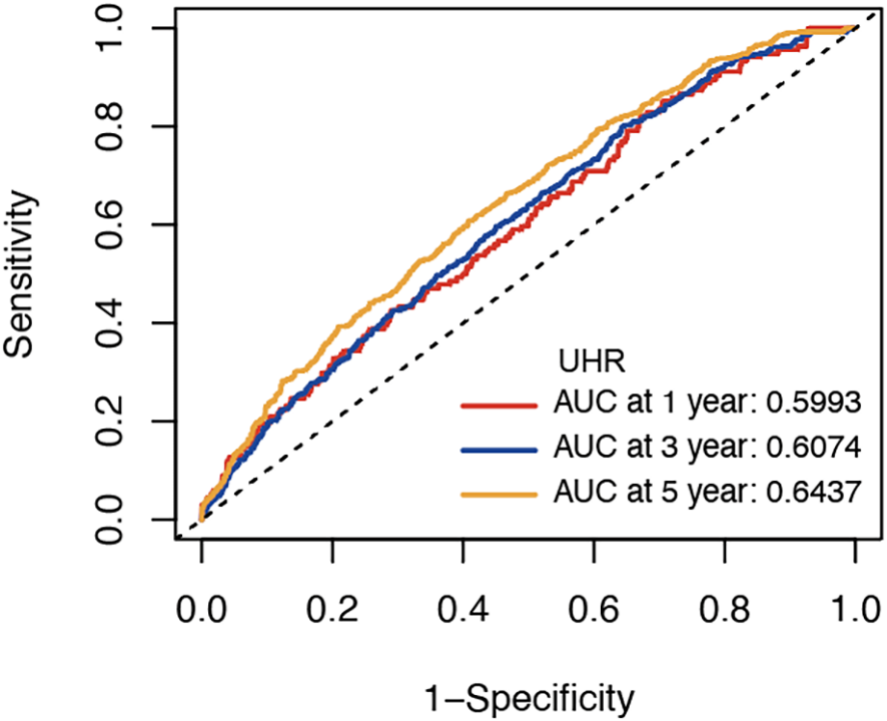
Time-dependent ROC curves for incident diabetes prediction by UHR.

In a cohort of 7525 elderly patients monitored for a median of 3.88 years [interquartile range (IQR): 2.87-4.86 years], 773 new cases of type 2 diabetes were reported. In fully adjusted Cox models, elevated baseline UHR was statistically linked to a greater hazard of type 2 diabetes after accounting for age, sex, lifestyle factors, and clinical indices (HR = 1.056, 95% CI 1.025-1.087, *P* < 0.001) (**Table 2**). Per 1-standard deviation (SD) increment in UHR, the hazard of incident diabetes increased by 15.6% (HR = 1.156, 95% CI 1.069-1.250, *P* < 0.001). When categorized into quartiles, individuals in the top UHR quartile exhibited a 1.7-fold higher risk relative to the bottom quartile in the fully adjusted model (HR = 1.700, 95% CI 1.385-2.088, *P* < 0.001;*P* for trend <0.001). The Kaplan-Meier analysis also supported these findings, showing a significantly greater cumulative risk in the highest UHR quartile group (*P* < 0.001) (**Figure 1**). Using a restricted cubic spline Cox model with threshold-effect analysis (**Table 3**), UHR showed a statistically significant nonlinear association with incident type 2 diabetes (*P* for nonlinear <0.001) (**Figure 2**). The estimated inflection point occurred at UHR = 4.478. Below this threshold, the risk increased markedly, whereas above the threshold the slope was shallower yet remained significant (**Table 3**). Using time-dependent ROC analysis (**Figure 3**), UHR demonstrated modest discrimination for incident diabetes, with corrected area under the curves (AUCs) of 59.93% at 1 year, 60.74% at 3 years, and 64.37% at 5 years. The predictive performance of UHR remains relatively stable over mid- to long-term follow-up, with a slight improvement at longer horizons.

**Table 2:** Results from COX regression analysis.

	Model 1	Model 2	Model 3
	HR (95%CI) *P* value		
Continuous			
SUA	1.003 (1.002, 1.004) <0.001	1.004 (1.003, 1.004) <0.001	1.001 (1.000, 1.002) 0.054
HDL-c	0.977 (0.971, 0.982) <0.001	0.975 (0.969, 0.980) <0.001	0.987 (0.981, 0.993) <0.001
UHR (μmol/L to mg/dL)	1.111 (1.091, 1.132) <0.001	1.126 (1.105, 1.148) <0.001	1.056 (1.025, 1.087) <0.001
UHR (μmol/L to mmol/L)	1.003 (1.002, 1.003) <0.001	1.003 (1.003, 1.004) <0.001	1.001 (1.001, 1.002) <0.001
Each SD increase	1.326 (1.262, 1.393) <0.001	1.375 (1.307, 1.446) <0.001	1.156 (1.069, 1.250) <0.001
Categories			
Q1	Reference	Reference	Reference
Q2	1.214 (0.990, 1.489) 0.0630	1.291 (1.050, 1.587) 0.015	1.140 (0.925, 1.405) 0.219
Q3	1.432 (1.176, 1.743) <0.001	1.608 (1.314, 1.966) <0.001	1.295 (1.054, 1.590) 0.014
Q4	2.034 (1.689, 2.450) <0.001	2.329 (1.915, 2.831) <0.001	1.700 (1.385, 2.088) <0.001
*P* for trend	<0.001	<0.001	<0.001

HR, Hazard ratio; SD, Standard deviation. 95% CI: 95% confidence interval. Model 1: no covariates adjusted. Model 2: adjusted for age, gender, smoking status, and alcohol consumption. Model 3: adjusted for age, gender, smoking status, alcohol consumption, hypertension, cardiovascular disease, BMI, FPG, ALT, AST, TG, LDL-c, BUN, Scr, and eGFR.

**Table 3:** Threshold effect of UHR on type 2 diabetes risk.

Model	HR (95% CI) *P* value
Total	1.056 (1.025, 1.087) <0.001
Breakpoint	4.478
HR1 (<4.478)	1.482 (1.188, 1.847) <0.001
HR2 (≥4.478)	1.038 (1.006, 1.071) 0.021
HR2/HR1	0.700 (0.558, 0.880) 0.002
*P* for logarithmic likelihood ratio	<0.001

HR, Hazard ratio. 95% CI, 95% confidence interval. Adjusted for age, gender, smoking status, alcohol consumption, hypertension, cardiovascular disease, BMI, FPG, ALT, AST, TG, LDL-c, BUN, Scr, and eGFR.

### 3.3 Subgroup and sensitivity analyses

**Figure 4:**
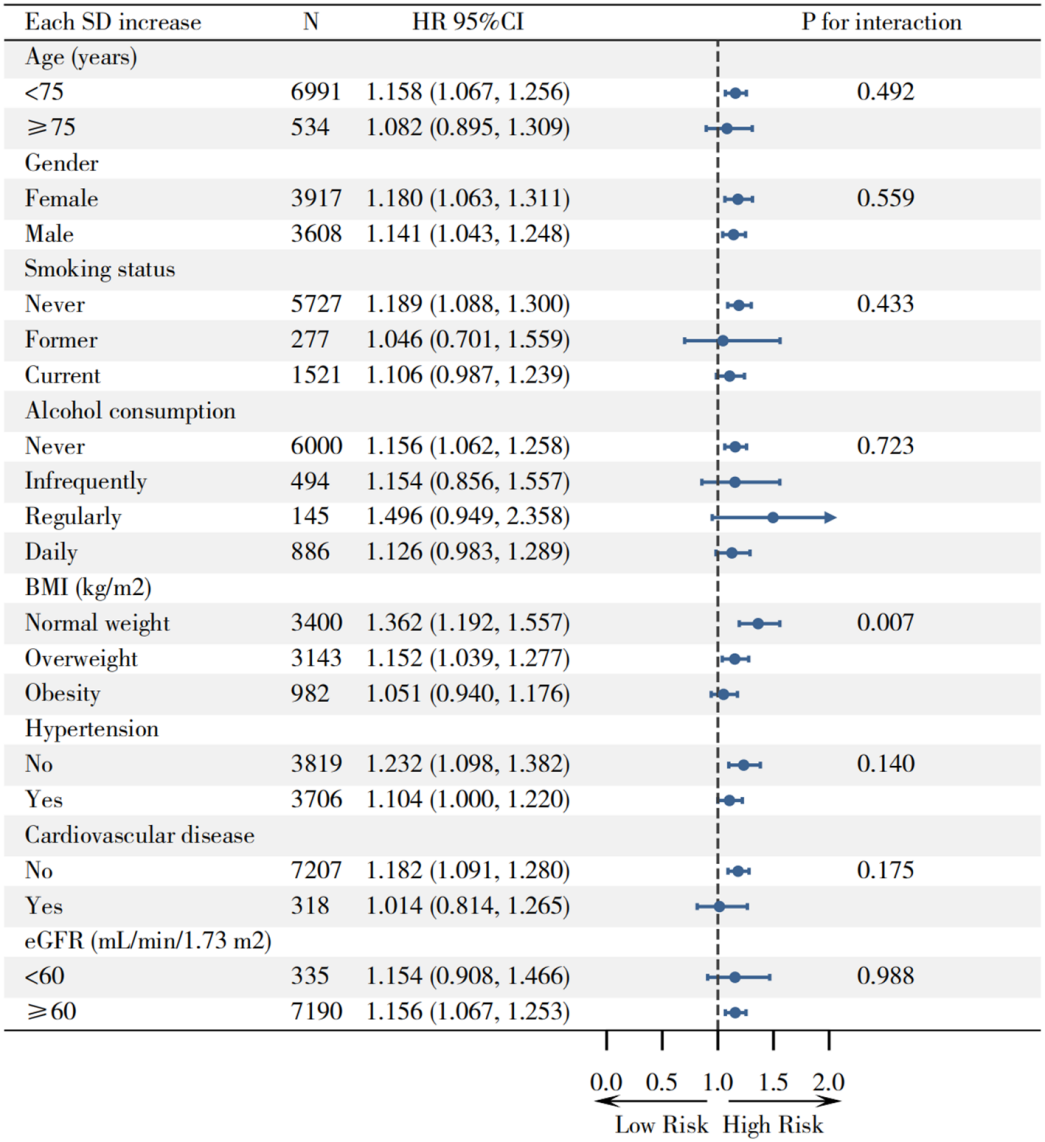
Subgroup analyses of the association between UHR and incident type 2 diabetes.

In prespecified subgroup analyses stratified by age, gender, smoking and alcohol consumption, BMI, hypertension, cardiovascular disease, and eGFR (**Figure 4**), a higher UHR remained positively associated with incident type 2 diabetes across most strata, with no evidence of effect modification by age, gender, smoking, alcohol intake, hypertension, cardiovascular disease, or eGFR (all *P* for interaction >0.05); however, a significant interaction was detected for BMI (*P* for interaction =0.007), whereby the UHR-diabetes association was strongest in non−obese participants-most pronounced at normal weight and attenuated in overweight-and weakest in those with obesity, indicating that elevated UHR confers greater relative risk at lower adiposity (**Figure 4**). To assess the robustness of our findings, we conducted sensitivity analyses by excluding participants with abnormal SUA (female>360 μmol/L; male>420 μmol/L) and HDL−c levels (male<40 mg/dL; female<50 mg/dL) and re−examined the association between UHR within the normal range and incident type 2 diabetes. The results were consistent with our primary analyses, reinforcing the reliability of the observed relationship (**Supplementary Materials**: **Supplementary Table S1**).

## 4 Discussion

To the best of our knowledge, this research represents the initial exploration of the link between UHR and the incidence of type 2 diabetes in elderly Chinese individuals. Our results indicate that elevated UHR levels correlate with an increased risk of developing type 2 diabetes, even within the normal range of UHR.

Previous research has highlighted the association between SUA and diabetes risk. Higher SUA levels have also been linked to metabolic syndrome, as reported by de Oliveira et al. in a cohort of apparently healthy Brazilian middle−aged men (17). Over a 15-year observation period, Krishnan et al. found that individuals with hyperuricemia were 1.87 times more likely to develop type 2 diabetes and 1.36 times more likely to exhibit insulin resistance (18). A meta-analysis based on 8 prospective cohort studies also indicated that high SUA levels are associated with an increased risk of type 2 diabetes in middle-aged and older adults, independent of known diabetes risk factors (19). A newly discovered function of HDL-c is their powerful antidiabetic effects (20). Increases in HDL-c are considered protective factors against insulin resistance (21). Many epidemiological studies have shown that low HDL-c levels are a significant risk factor for diabetes. Data from the Framingham Offspring Study suggests that the risk of developing type 2 diabetes decreases by about 4% for every 1 mg/dL increase in HDL-c over a 7-year follow-up period (22). A study of over 5 million nondiabetic Korean adults found that low HDL-c levels were associated with a higher risk of developing diabetes over a span of 5.1 years, particularly in those with fluctuating HDL-c levels (23). UHR reflects the combined effects of metabolic disorders and inflammation on the risk of diabetes (24). Our findings extend previous research on UHR and glucose metabolism. In patients with established type 2 diabetes, Zhou and Xu reported that UHR outperformed SUA or HDL−c alone in identifying insulin resistance. However, these studies have mainly focused on insulin resistance or prevalent diabetes in mixed−age populations (24). No longitudinal study has examined whether baseline UHR predicts incident type 2 diabetes among community−dwelling older adults or evaluated potential nonlinear threshold effects and the time−dependent predictive performance of UHR for future diabetes. Our study addresses these gaps by demonstrating a prospective association between UHR and diabetes risk in an elderly cohort and by assessing both nonlinear dose-response patterns and time−varying predictive value.

Several potential mechanisms might help explain the connection between UHR and diabetes risk. Animal studies have shown that fructose-induced hyperuricemia is linked to the development of metabolic syndrome, and lowering uric acid levels can improve this condition (25). Hyperuricemia has been proven to cause endothelial dysfunction and reduce nitric oxide production (26). Reduced nitric oxide can decrease glucose uptake in skeletal muscles in response to insulin, leading to insulin resistance and diabetes (27, 28). Hyperuricemia is associated with oxidative stress and inflammation, playing a significant role in the pathogenesis of diabetes (29, 30). HDL-c has been discovered to enhance endothelial health by increasing nitric oxide (NO) production and inhibiting pathways that cause endothelial cell death and vascular inflammation (31). HDL-c can protect pancreatic beta cells from oxidative stress and inflammation, maintaining normal insulin secretion (32–34). HDL-c helps enhance insulin sensitivity, promoting glucose uptake by skeletal muscle and other tissues, thereby lowering blood sugar levels (35, 36). In the RCS analysis, we observed a non−linear association between UHR and incident type 2 diabetes, with an inflection point around UHR = 4.478. Below this value, diabetes risk increased more steeply with higher UHR, while above it the slope was flatter but still positive. This pattern is compatible with the joint effects of uric−acid–related oxidative stress and inflammation and the loss of HDL−related antioxidative capacity. Nevertheless, the estimated threshold should be interpreted cautiously. Most participants in our cohort had UHR values between about 2 and 6, and the inflection point derived from spline−based maximum likelihood estimation may therefore be partly driven by the underlying data density rather than a true biological cut−off. Thus, the threshold should be regarded as a statistical indicator of risk acceleration instead of a definitive clinical threshold, and confirmation in other populations and mechanistic studies is warranted.

However, this investigation still has several limitations. Firstly, the findings of this retrospective cohort study are applicable solely to the elderly population in Kunshan, China. Caution should be taken when generalizing these results to other regions or age groups. Several important potential confounders-such as physical activity, dietary patterns, measures of central obesity, socioeconomic status, and use of relevant medications -were not comprehensively captured in our dataset and therefore could not be fully adjusted for, which may have led to residual confounding. Thirdly, diabetes ascertainment relied on ICD−10 codes and FPG measurements from routine health check−ups, without systematic assessment of oral glucose tolerance test (OGTT) or glycated hemoglobin A1c (HbA1c). We also could not verify repeat FPG testing or additional confirmatory tests for each participant, and some individuals with transient hyperglycemia may therefore have been misclassified as having diabetes. Moreover, the time−dependent AUCs of 0.60-0.64 indicate only modest discrimination, which is insufficient for stand−alone screening or diagnosis. Taken together, these findings suggest that UHR might be more appropriately used as a simple marker that complements established metabolic risk factors in future multivariable risk models, rather than as an independent screening or diagnostic tool. Lastly, this study only considered the baseline UHR levels and did not account for the impact of UHR fluctuations during follow-up on the risk of developing type 2 diabetes.

## 5 Conclusion

In conclusion, this study of community-dwelling older adults in a single Chinese city identified a positive association between UHR and type 2 diabetes risk. Monitoring UHR may aid in assessing diabetes risk in the elderly; however, the generalizability of these findings to younger, rural, or more ethnically diverse populations remains to be determined.

## Data Availability

The data analyzed in this study is subject to the following licenses/restrictions: The healthcare data analyzed in this study were obtained from the primary medical center in Kunshan and anonymized to protect privacy. Summary data supporting the study’s findings are available upon request from the corresponding author. Requests to access these datasets should be directed to lubing_0527@163.com.

## Supplementary material

Supplementary Table 1 Sensitivity analyses restricting to normal SUA and HDL−C ranges.
