# Supplementary material for: Association of the uric acid-to-HDL cholesterol ratio with incident type 2 diabetes in community-dwelling older adults in China: a retrospective cohort study
Source: Front Endocrinol (Lausanne). 2026 Jan 6;16:1720947. doi: 10.3389/fendo.2025.1720947 (PMC12815726; doi:10.3389/fendo.2025.1720947)
Supplement: Supplementary Table 1 — Sensitivity analyses restricting to normal SUA and HDL−C ranges. [file Table1.docx]

| **Table S1** Sensitivity Analyses Restricting to Normal SUA and HDL‑C Ranges. | | | |
| --- | --- | --- | --- |
|  | Model 1 | Model 2 | Model 3 |
|  | HR (95%CI) ***P*** value | | |
| Continuous | | | |
| SUA | 1.001 (1.000, 1.003) 0.105 | 1.002 (1.000, 1.004) 0.017 | 1.001 (0.999, 1.003) 0.311 |
| HDL-c | 0.981 (0.972, 0.990) <0.001 | 0.977 (0.967, 0.987) <0.001 | 0.983 (0.972, 0.993) 0.002 |
| UHR  (μmol/L to mg/dL) | 1.121 (1.053, 1.195) <0.001 | 1.202 (1.114, 1.297) <0.001 | 1.124 (1.032, 1.223) 0.007 |
| UHR  (μmol/L to mmol/L) | 1.003 (1.001, 1.005) <0.001 | 1.005 (1.003, 1.007) <0.001 | 1.003 (1.001, 1.005) 0.007 |
| Each SD increase | 1.199 (1.084, 1.325) <0.001 | 1.338 (1.186, 1.508) <0.001 | 1.202 (1.052, 1.375) 0.007 |
| Categories | | | |
| Q1 | Reference | Reference | Reference |
| Q2 | 1.455 (1.057, 2.003) 0.022 | 1.545 (1.120, 2.130) 0.008 | 1.464 (1.054, 2.033) 0.023 |
| Q3 | 1.648 (1.206, 2.253) 0.002 | 1.875 (1.362, 2.581) <0.001 | 1.771 (1.273, 2.464) <0.001 |
| Q4 | 1.788 (1.315, 2.432) <0.001 | 2.340 (1.652, 3.314) <0.001 | 1.787 (1.228, 2.600) 0.002 |
| ***P*** for trend | <0.001 | <0.001 | <0.001 |
| HR: Hazard ratio; SD: Standard deviation  95% CI: 95% confidence interval.  Model 1: no covariates adjusted.  Model 2: adjusted for age, gender, smoking status, and alcohol consumption.  Model 3: adjusted for age, gender, smoking status, alcohol consumption, hypertension, cardiovascular disease, BMI, FPG, ALT, AST, TG, LDL-c, BUN, Scr, and eGFR | | | |
